# Supplementary figures and images for: Altered mucins and aquaporins indicate dry eye outcome in patients undergoing Vitreo-retinal surgery
Source: PLoS One. 2020 May 21;15(5):e0233517. doi: 10.1371/journal.pone.0233517 (PMC7241722; doi:10.1371/journal.pone.0233517)

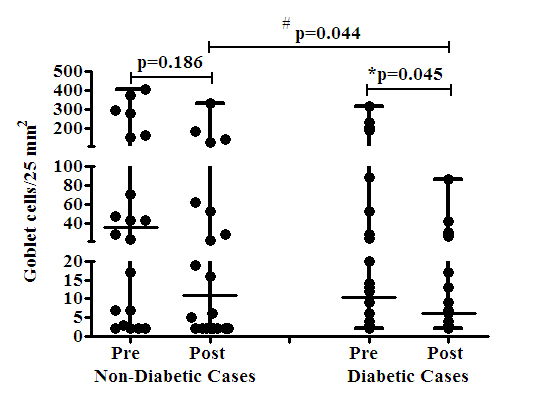

Supplement: S1 Fig — Non-DM (n = 20) and DM cases (n = 24). *p<0.05 is the comparison between pre and post VR surgery; # p < 0.05 is the comparison between post-VR surgery in non-DM vs DM. (JPG) [file pone.0233517.s001.jpg]

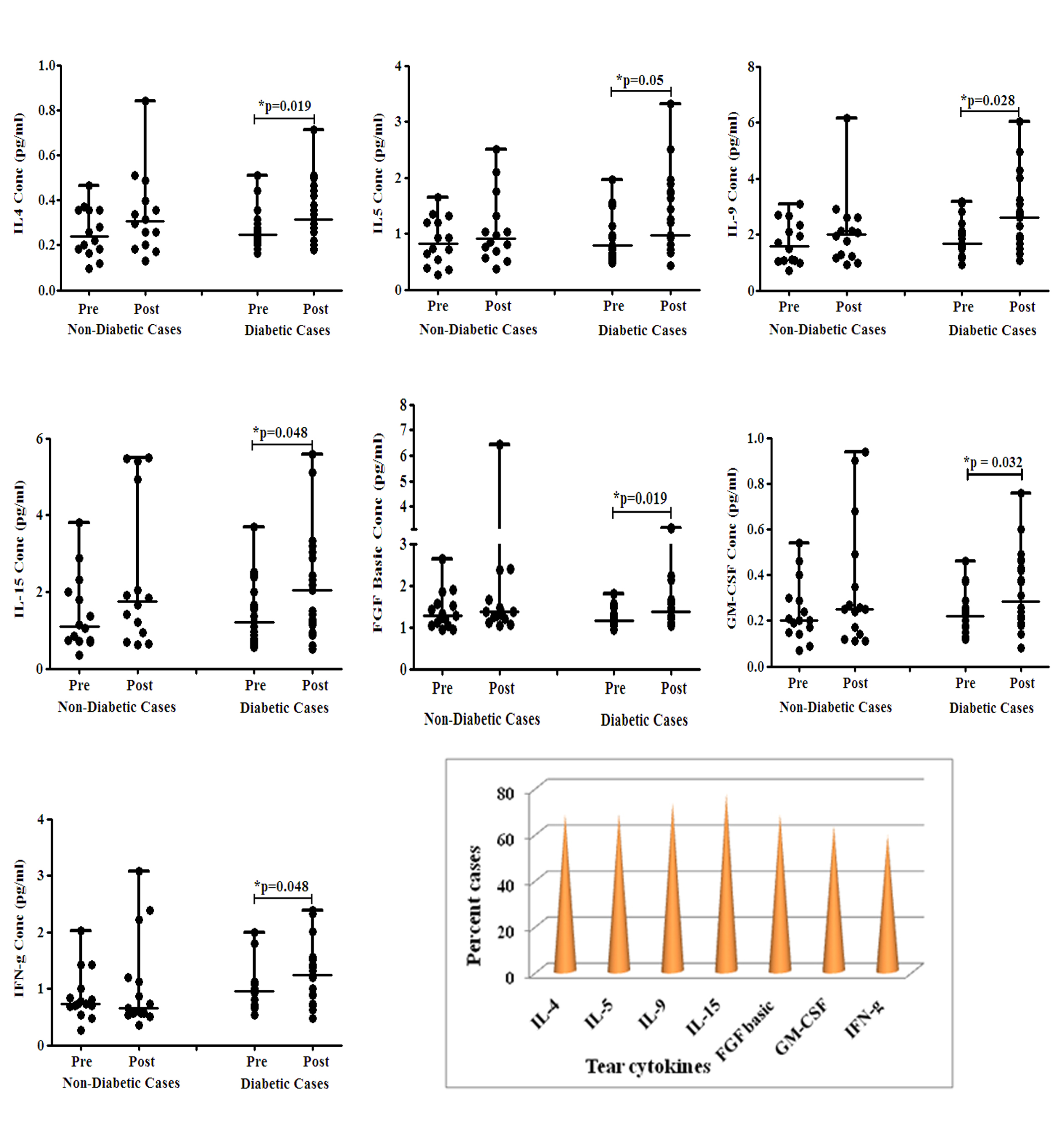

Supplement: S2 Fig — The bar graph depicting percentage of cases with significantly up-regulated tear cytokines after VR surgery. (n = 24); *p<0.05 is the comparison between pre and post VR surgery. (JPG) [file pone.0233517.s002.jpg]
