# Supplementary material for: Altered mucins and aquaporins indicate dry eye outcome in patients undergoing Vitreo-retinal surgery
Source: PLoS One. 2020 May 21;15(5):e0233517. doi: 10.1371/journal.pone.0233517 (PMC7241722; doi:10.1371/journal.pone.0233517)
Supplement: S1 Table — p-value is a comparison between pre and the corresponding post-VR surgery tear cytokine. *p<0.05 and **p<0.01. Statistically significant p-values are given in bold. Sample size = 36. (DOCX) [file pone.0233517.s003.docx]

**S1 Table. Tear cytokines profile pre and post vitreo-retinal surgery**

| **S.**  **No** | **Tear Cytokines** | **Pre-surgery**  **(n=36)** | **Post-surgery**  **(n=36)** | **p-value** | **Percent cases showing**  **≥2 fold change** | | |
| --- | --- | --- | --- | --- | --- | --- | --- |
|  |  | **Mean ± SD/(Median)**  **pg/mL** | **Mean ± SD/(Median)**  **pg/mL** |  | **Increased %** | **Decreased %** | **No change %** |
| 1 | IL1b | 0.29 ± 0.15(0.22) | 2.65 ± 8.5 (0.34) | 0.099 | 67 | 22 | 11 |
| 2 | IL1RA | 6156 ± 3686 (5227) | 8857 ± 6663 (6495) | **0.046** | 64 | 28 | 8 |
| 3 | IL2 | 0.52 ± 0.14 (0.5) | 0.63 ± 0.31 (0.58) | **0.05** | 69 | 28 | 3 |
| 4 | IL4 | 0.26 ± 0.1 (0.25) | 0.35 ± 0.15 (0.32) | **0.008** | 67 | 25 | 8 |
| 5 | IL5 | 0.9 ± 0.41 (0.79) | 1.2 ± 0.69 (0.97) | **0.027** | 67 | 30 | 3 |
| 6 | IL6 | 0.31 ± 0.13 (0.28) | 0.47 ± 0.43 (0.33) | **0.044** | 64 | 31 | 5 |
| 7 | IL7 | 32.11 ± 19.7 (28.74) | 42.08 ± 32.65 (37.14) | 0.127 | 61 | 33 | 6 |
| 8 | IL8 | 24.47 ±24.88 (14.65) | 50.65 ± 137.44 (17.72) | 0.265 | 67 | 33 | 0 |
| 9 | IL9 | 1.83 ± 0.71 (1.66) | 2.44 ± 1.32 (2.12) | **0.019** | 58 | 31 | 11 |
| 10 | IL10 | 1.05 ± 0.45 (1.02) | 2.22 ± 5.8 (1.21) | 0.232 | 67 | 33 | 0 |
| 11 | IL12(p70) | 1.09 ± 0.38 (1.04) | 1.31 ± 0.94 (1.14) | 0.184 | 61 | 31 | 8 |
| 12 | IL13 | 0.87 ± 0.88 (0.66) | 1.27 ± 2.31 (0.76) | 0.333 | 67 | 30 | 3 |
| 13 | IL15 | 1.44 ± 0.87 (1.14) | 2.26 ± 1.61 (1.85) | **0.01** | 78 | 19 | 3 |
| 14 | IL17 | 1.89 ± 0.69 (1.74) | 2.57 ± 2.52 (2.02) | 0.125 | 72 | 25 | 3 |
| 15 | Eotaxin | 2.21 ± 1.51 (1.97) | 2.72 ± 2.07 (1.88) | 0.26 | 53 | 39 | 8 |
| 16 | FGF | 1.29 ± 0.35 (1.21) | 1.62 ± 0.94 (1.38) | **0.05** | 64 | 33 | 3 |
| 17 | G-CSF | 7.59 ± 4.95 (6.27) | 10.3 ± 12.85 (6.71) | 0.243 | 64 | 36 | 0 |
| 18 | GM-CSF | 0.24 ± 0.11 (0.21) | 0.34 ± 0.22 (0.26) | **0.016** | 69 | 25 | 6 |
| 19 | IFNg | 0.94 ± 0.39 (0.92) | 1.25 ± 0.73 (1.16) | **0.029** | 64 | 33 | 3 |
| 20 | IP10 | 27214 ±22069 (20821) | 26539 ±20862 (20010) | 0.899 | 47 | 45 | 8 |
| 21 | MCP1 | 20.96 ± 23.54 (9.65) | 22.97 ± 27.91 (12.16) | 0.721 | 47 | 42 | 11 |
| 22 | MIP1a | 4406 ± 4202 (2496) | 5153 ± 16258 (2019) | 0.791 | 56 | 36 | 8 |
| 23 | PDGF-bb | 11.67 ± 5.3 (12.37) | 15.03 ± 8.57 (13.01) | **0.05** | 61 | 33 | 6 |
| 24 | MIP1b | 1.29 ± 2.23 (0.61) | 2.07 ± 2.25 (1.33) | 0.146 | 67 | 28 | 5 |
| 25 | RANTES | 26.86 ± 27.78 (16.3) | 30.84 ± 34.87 (22.38) | 0.594 | 58 | 42 | 0 |
| 26 | TNFα | 7.28 ± 2.23 (6.86) | 8.42 ± 2.74 (7.57) | 0.06 | 61 | 33 | 6 |
| 27 | VEGF | 42.11 ±39.62 (34.46) | 67.21 ± 118.15 (36.94) | 0.231 | 67 | 33 | 0 |

Statistically significant cytokines: p-value (*p<0.05 and **p<0.01), p-value is a comparison between pre and the corresponding post-VR surgery tear cytokine. Statistically significant p-values are given in bold.
