# Supplementary material for: Altered mucins and aquaporins indicate dry eye outcome in patients undergoing Vitreo-retinal surgery
Source: PLoS One. 2020 May 21;15(5):e0233517. doi: 10.1371/journal.pone.0233517 (PMC7241722; doi:10.1371/journal.pone.0233517)
Supplement: S2 Table — p-value is a comparison between pre and the corresponding post-VR surgery tear cytokine. *p<0.05, **p<0.01 and ***p<0.001. Statistically significant p-values are given in bold. Sample size = 36. (DOCX) [file pone.0233517.s004.docx]

**S2 Table. Cytokines profiling based on vitreo-retinal surgeries**

| **S.No** | **Parameters** | **360^o^ Conjunctival opening (n=3)** | **Conjunctival non opening (n=41)** | **Suture (n=16)** | **Non-suture (n=28)** | **Hemorrhage (n=23)** | **Non-Hemorrhage (n=21)** |
| --- | --- | --- | --- | --- | --- | --- | --- |
|  |  |  |  |  |  |  |  |
| 1 | **IL1b** | 0.232 | **0.05** | 0.217 | 0.119 | 0.125 | 0.198 |
| 2 | **IL1ra** | 0.886 | **0.002** | 0.084 | **0.006** | **0.003** | 0.086 |
| 3 | **IL2** | 0.474 | **0.012** | 0.119 | **0.018** | **0.026** | 0.073 |
| 4 | **IL4** | 0.989 | **0.0001** | **0.001** | **0.007** | **0.0001** | **0.05** |
| 5 | **IL5** | 0.85 | **0.0001** | **0.008** | **0.011** | **0.003** | **0.05** |
| 6 | **IL6** | 0.638 | **0.01** | 0.178 | **0.027** | **0.075** | **0.05** |
| 7 | **IL7** | 0.699 | **0.01** | 0.086 | **0.026** | **0.03** | 0.146 |
| 8 | **IL8** | 0.399 | 0.225 | 0.84 | 0.193 | 0.22 | 0.755 |
| 9 | **IL9** | 0.828 | **0.001** | **0.003** | **0.024** | **0.001** | 0.12 |
| 10 | **IL10** | 0.611 | 0.129 | 0.273 | **0.005** | **0.01** | 0.227 |
| 11 | **IL12(p70)** | 0.905 | 0.067 | 0.298 | **0.042** | 0.255 | 0.151 |
| 12 | **IL13** | 0.719 | 0.167 | 0.183 | 0.874 | **0.01** | 0.411 |
| 13 | **IL15** | 0.781 | **0.0001** | **0.004** | **0.001** | **0.0001** | **0.02** |
| 14 | **IL17** | 0.901 | **0.04** | 0.193 | **0.011** | **0.001** | 0.153 |
| 15 | **Eotaxin** | 0.817 | 0.079 | 0.203 | 0.252 | 0.1 | 0.498 |
| 16 | **FGF** | 0.818 | **0.02** | 0.118 | **0.04** | **0.021** | 0.133 |
| 17 | **G-CSF** | 0.831 | 0.145 | 0.291 | 0.366 | 0.202 | 0.456 |
| 18 | **GM-CSF** | 0.708 | 0.094 | 0.246 | **0.002** | **0.004** | 0.187 |
| 19 | **IFNg** | 0.764 | 0.111 | 0.217 | **0.003** | **0.006** | 0.213 |
| 20 | **IP10** | 0.617 | 0.695 | 0.544 | 0.4 | 0.76 | 0.922 |
| 21 | **MCP1** | 0.889 | 0.307 | 0.742 | 0.352 | 0.208 | 0.695 |
| 22 | **MIP1a** | 0.645 | 0.815 | 0.515 | **0.03** | 0.491 | **0.05** |
| 23 | **PDGF-bb** | 0.712 | **0.001** | **0.009** | **0.03** | **0.001** | 0.3 |
| 24 | **MIP1b** | 0.58 | 0.072 | 0.912 | **0.008** | 0.268 | 0.06 |
| 25 | **RANTES** | 0.968 | 0.455 | 0.62 | 0.577 | 0.671 | 0.505 |
| 26 | **TNFa** | 0.126 | **0.008** | **0.05** | 0.084 | 0.053 | 0.127 |
| 27 | **VEGF** | 0.63 | 0.125 | 0.233 | 0.143 | **0.03** | 0.247 |

Statistically significant cytokines: p-value (*p<0.05, **p<0.01 and ***p<0.001), p-value is a comparison between pre and the corresponding post-VR surgery tear cytokine. Statistically significant p-values are given in bold.
