# Supplementary material for: Altered mucins and aquaporins indicate dry eye outcome in patients undergoing Vitreo-retinal surgery
Source: PLoS One. 2020 May 21;15(5):e0233517. doi: 10.1371/journal.pone.0233517 (PMC7241722; doi:10.1371/journal.pone.0233517)
Supplement: Attachment — Submitted filename: Response to Reviewers.doc [file pone.0233517.s005.doc]

| S.No. | **Reviewer 1 Comments** | **Reply** |
| --- | --- | --- |
| 1. | Dry eye disease (DED) is clearly a multifactorial condition yet it is wholly a clinical diagnosis. The tolerance of a given individual to the studied molecular changes may or may not be harbingers of future DED. | The clinical diagnosis of dry eye is based on cumulative score with limitations of sensitivity /specificity of single tests. Tear Osmolality is considered the best but is still limited by high variability.  The study interpretations were based on the statistics ie number of cases that showed molecular changes indicative of DED.  However, we do agree that the validation in a larger cohort based on follow-up studies of the surgical intervention cases can be conclusive.  This is included in the conclusion |
| 2. | The incorporation of DM patients, those undergoing retina detachment procedures and the newer trans conjunctival vitrectomy procedures in a single opus is confusing. . Each cohort should be treated separately. | The inclusion criterion was vitreoretinal surgery which included diabetic as well as non diabetic patients undergoing the surgery for different indications. Two types of surgeries were performed viz. scleral buckle in 3 patients and transconjunctival suture less pars plana vitrectomy (PPV) in the rest. The PPV is the more commonly performed surgery for vitreoretinal diseases the world over.  We agree with the reviewer. But even though the study included these different cohorts, we have analysed them separately. Table 2 gives the details in different groups. We feel that the inclusion of different cohorts has added value to the study (by giving comparative data). |
| 3. | The use of topical steroids postoperatively was not discussed | This has now been added in the methods section. |
| 4. | Were there significant variations in administration or was a single protocol utilized. | There were no variations and the same fixed protocol was followed for all the patients, pre, intra and post-surgery. |
|  |  |  |
|  | **Reviewer 2 Comments** |  |
| 1. | This is an interesting article relating the occurrence of Dry eye syndrome after vitrectomy. The methods performed in this manuscript are accurate and reasonable. All the conditions for inclusion and exclusion are done correctly. However the conclusions made in this article seem a little over-reaching. | Conclusion is now modified based on the study limitations and is less emphatic. |
| 2. | While it is true that multiple case studies have shown the occurrence of DES with vitrectomy, the results from this study do not seem to be as conclusive as one would like to see. | This study supports the DES occurrence during victroctomy based on molecular changes rather than clinical signs and symptoms.  The limitation of the study is sample size is less after segregating based on the surgery type or presence and absence of type 2DM. Secondly, no longitudinal follow-up was done. |
| 3. | The pros of this manuscript are the recommendations made to Diabetic patients.  It is also very interesting to see that mucin secretion and GCD go down significantly after the surgical procedure.  The cons include low and under-explanation of the results and almost complete absence of discussion of the results. | Results on mucin /goblet cell density and aquaporins are now mentioned in detail.  Discussion is now improved with elaboration on mucin / goblet cell density and aquaporins. |
| 4. | I would recommend the authors to reconsider how they portray the results better in an intelligible fashion. | The results have been portrayed better now. |
| 5. | I do not understand the purpose of Figure 4. It is neither very informative nor self explanatory nor the authors have even attempted to explain whats going on in this figure. I would infact recommend the complete removal of this figure unless a better explanation is made. | Figure.4 is removed |
| 6. | Another question I had about the article is whether cytokines are elevated after any ocular surgery? | This is now discussed in the Discussion section paragraph 3 as below:  A disturbance of postoperative inflammation response after laser treatment when compared with control group (Manual cataract surgery) is reported after 30 days (Chen et al., 2015).  After 7 days of femtosecond laser-assisted cataract surgery rise in proinflammatory cytokines in the aqueous humor was observed (Favuzza et al., 2019).  Our own previous study report on cytokine profile in tears from Chronic SJS cases who underwent mucous membrane grafting (MMG) on follow up between 2-3 months showed characteristic changes [pre-MMG and post-MMG levels of tear cytokines) which is in response to the mucous membrane graft. (Gurumoorthy et al., 2018).  Thus, the type of tissue and surgical insult as well as time factor determine the cytokine status apart from the age/sex and diabetic status of the patient |
| 7. | Does any surgery cause cytokine increase or does this particular surgery casue it? | Surgical trauma does alter the immune homeostasis with changes in pro- as well as anti-inflammatory cytokines and seems to be important in planning the therapeutic strategies, post-surgery (Ni et al., 2006).  This cellular response may vary with tissue type though there can be overlap in some of the cytokine/chemokine/growth factors expression. It can also vary with patient status such as immunocompromised patient (Lin et al., 2000)  This is now included in the discussion: paragraph 3 |
| 8. | May be some information regarding this in the discussion section can carve out a better explanation about the particular vitrectomy as a responsible factor for DES. | Acute changes are reported post-surgery. But we have done a follow up after 8 weeks. This is now included in discussion.  Characteristic cytokine changes observed in various type of surgical insults is now included a supp table 2.  Discussion states as now follows:  “Though not supported by clinical signs and symptoms, lowered GCD and lowered MUC5AC indicates dry eye in DM cases who underwent VR surgery especially in the vitreous haemorrhage, non-suture and conjunctival non-opening group. This is further supported by the fact that the maximal cytokine response was also seen in these groups (supp table 2)”. |
| 9. | The authors may also consider explaining the acronyms for the genes better in the manuscript. This would increase the readability of the paper. | This is now included in the abstract / introduction, when mentioned for the first time. |
